# Hair dehydroepiandrosterone sulfate as biomarker of employees’ well-being? A longitudinal investigation of support, resilience, and work engagement during COVID-19 pandemic

**DOI:** 10.3389/fpsyg.2024.1337839

**Published:** 2024-03-20

**Authors:** Damiano Girardi, Laura Dal Corso, Elvira Arcucci, Murat Yıldırım, Isabella Pividori, Alberto Prandi, Alessandra Falco

**Affiliations:** ^1^FISPPA Section of Applied Psychology, University of Padua, Padua, Italy; ^2^Department of Psychology, Agri Ibrahim Cecen University, Ağrı, Türkiye; ^3^Department of Psychology, Nottingham Trent University, Nottingham, United Kingdom; ^4^Department of Social and Educational Sciences, Lebanese American University, Beirut, Lebanon; ^5^Department of Agricultural, Environmental and Animal Sciences, University of Udine, Udine, Italy

**Keywords:** supervisor support, resilience, work engagement, hair dehydroepiandrosterone sulfate, biomarker, COVID-19, organizational well-being

## Abstract

**Introduction:**

Building on the motivational process of the job demands-resources (JD-R) theory, in the current research we investigated the longitudinal association between supervisor support/resilience as job/personal resources, work engagement (WE) and hair dehydroepiandrosterone sulfate, or DHEA(S), as a possible biomarker of employees’ well-being.

**Methods:**

In the context of the COVID-19 pandemic, 122 workers completed two self-report questionnaires (i.e., psychological data): the former at Time 1 (T1) and the latter three months afterwards, at Time 2 (T2). Participants also collected a strand of hair (i.e., biological data) at T2.

**Results:**

Results from path analysis showed that both SS and resilience at T1 were positively related to WE at T2, which, in its turn, was positively related to hair DHEA(S) at T2. Both SS and resilience at T1 had a positive indirect effect on hair DHEA(S) at T2 through WE at T2, which fully mediated the association between job/personal resources and hair DHEA(S).

**Discussion:**

Overall, results are consistent with the motivational process of the JD-R. Furthermore, this study provides preliminary evidence for the role of hair DHEA(S) as a biomarker of WE, a type of work-related subjective well-being that plays a central role in the motivational process of the JD-R, leading to favorable personal and organizational outcomes. Finally, the article outlines practical implications for organizations and professionals to foster WE within the workplace.

## 1 Introduction

In recent years, enhancing the quality of the work environment has been acknowledged as a policy priority (Murtin et al., 2022) as well as a key factor in fostering employees’ psychosocial well-being (Siegrist et al., 2007). For example, one of the goals of the Sustainable Development Agenda (Goal 8) is to “promote inclusive and sustainable economic growth, employment and decent work for all,” with decent work referring to opportunities for everyone to get work that delivers—among others—a fair income, security in the workplace, and, interestingly, better prospects for personal development and social integration. Work engagement (WE)—a “positive, fulfilling, work-related state of mind that is characterized by vigor, dedication, and absorption” (Schaufeli et al., 2002, p. 74)—is often identified as one of the crucial outcome indicators of sustainable work because, by being associated with job satisfaction and performance, it establishes the conditions for the worker to enjoy and remain at work (Eiffe, 2021).

Not surprisingly, in the last few years scholars (Mazzetti et al., 2021) and practitioners (Gallup, 2022) have devoted increasing attention to WE, since previous research suggests that fostering WE may lead to favorable outcomes for both organizations and employees, in terms of productivity and well-being (Bakker et al., 2023). For example, it has been shown that, on the one hand, organizations can achieve meaningful business outcomes—including customer satisfaction, productivity, profit, and reduced employee turnover—through high levels of WE among employees (Harter et al., 2002). On the other hand, prior studies outlined WE as an active, positive type of work-related subjective well-being (SWB) (Oerlemans and Bakker, 2011; Lesener et al., 2020) that is characterized by fulfillment, energy, and strong identification with one’s work. Interestingly, this makes WE different from job burnout, a negative type of work-related SWB that is characterized by the opposite, that is, low energy and low identification with one’s work (Oerlemans and Bakker, 2011). Summarizing, high levels of WE are generally beneficial for both organizations and employees.

Recently, the COVID-19 pandemic has posed a serious challenge to organizations and managers, called to support employees’ safety and well-being—also in terms of WE—in a period characterized by uncertainty and adversities (Eurofound, 2022). In fact, since the beginning of the COVID-19 crisis in early 2020, firms had to face prolonged business closures to reduce the spread of the new Coronavirus, reorganize their work processes (e.g., massive adoption of telecommuting/smart working, new measures to protect employees’ health and safety), as well as navigate economic uncertainty (International Labour Organization, 2021), all of which had significant implications for organizational effectiveness and sustainability. Clearly, this had negative consequences for workers, in terms of heavy workload and work-pace, increased job insecurity and social isolation, reduced work-life balance as well as layoffs, pay cuts, and furloughs (International Labour Organization, 2020; Eurofound, 2022), which have represented a relevant threat to employee WE (De-la-Calle-Durán and Rodríguez-Sánchez, 2021). Not surprisingly, data from working population surveys showed that less than half of the European workers (i.e., 42%) reported a high level of engagement at work in 2021, although with differences across gender, age, and occupational sectors (Eurofound, 2022). Similarly, it was estimated that 21% of employees globally were engaged at work in 2021, with the cost of low engagement to the global economy estimated at US$ 7.8 trillion (11% of gross domestic product globally) (Gallup, 2022). Therefore, studies investigating factors that can sustain WE, as well as its psycho-physiological underpinnings, are extremely relevant, with implications for both researchers and practitioners.

In this perspective and building on the job demands-resources (JD-R) theory (Bakker and Demerouti, 2017; Bakker et al., 2023), in this paper we examined the role of an organizational and an individual factor—namely supervisor support (SS) and resilience—in promoting WE during the COVID-19 pandemic. According to the JD-R theory, these factors are considered as job resources (JRs) and personal resources (PRs), both of which are expected to play a central role in fostering WE (Mazzetti et al., 2021; Bakker et al., 2023). Furthermore, in this study we concentrated on measurement of dehydroepiandrosterone sulfate (DHEA(S)) in hair, considered as a possible biomarker of employees’ well-being (Qiao et al., 2017) based on its association with JRs/PRs and WE, in line with the motivational process of the JD-R theory. One advantage of hair measurement is that it enables retrospective evaluation of cumulative DHEA(S) concentrations from baseline to follow-up (i.e., throughout the observation period).

Summarizing, in the current study we investigated longitudinally the association between SS/resilience (as JRs/PRs), WE, and a possible biomarker of employees’ well-being, namely hair DHEA(S). In our view, our contribution to literature is threefold. First, by addressing the call by Bakker and Demerouti (Bakker and Demerouti, 2017, p. 277) to “further explore the psychological and physiological processes involved in the health impairment and motivational processes in JD-R theory,” this study aims to shed light on the cumulative physiological response associated with JRs/PRs and WE over time. Next, by focusing on hair DHEA(S), this research contributes to identify a panel of possible biomarkers of well-being at work—similar to the composite allostatic load index for chronic stress (Juster et al., 2010; Polacchini et al., 2018)—with possible theoretical and practical implications. Finally, by adopting a longitudinal, multi-method design that integrates different measurement methods (Falco et al., 2012; Podsakoff et al., 2012), we aim to make a substantive contribution to the identification of possible antecedents/consequences of WE, as previous research has largely been based on cross-sectional, self-report data (Crawford et al., 2010; Christian et al., 2011), although with some notable exceptions (see for example Lesener et al., 2020). The remaining sections are organized as follows: firstly, we briefly describe the JD-R theory as the overarching theoretical framework for the study. Next, we describe the hypotheses of the study and the underlying theoretical mechanisms. In doing so, we focus on hair DHEA(S) and its role as a possible biomarker of employees’ well-being.

**Figure 1:**
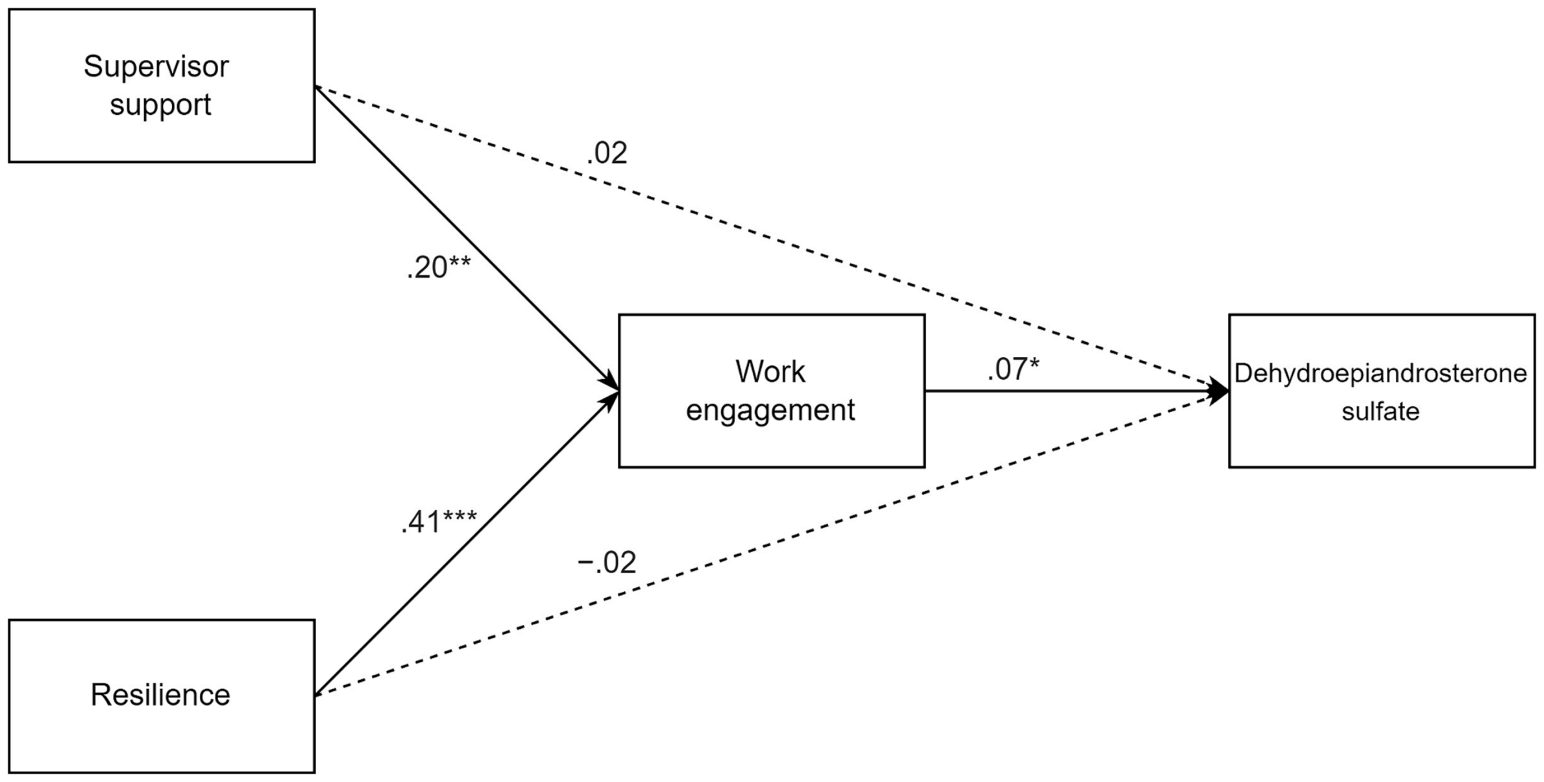
Path analysis model of associations between hair dehydroepiandrosterone sulfate, work engagement, supervisor support, and resilience (*N* = 122). Dehydroepiandrosterone sulfate values were log-transformed prior to data analyses. ^*^p < 0.05, ^**^p < 0.01, ^***^p < 0.001.

### 1.1 The job demands-resources theory

This study builds on the JD-R theory (Demerouti et al., 2001; Bakker and Demerouti, 2017), a flexible theoretical framework that is extremely valuable to understand employee’s well-being and performance across different occupational contexts (Bakker et al., 2023). Specifically, according to the JD-R, job characteristics can be classified either as job demands (JDs) or JRs. First, JDs are those “physical, psychological, social, or organizational aspects of the job that require sustained physical and/or psychological effort and are therefore associated with certain physiological and/or psychological costs” (Bakker and Demerouti, 2017, p. 274) and include for example workload or job insecurity. Next, JRs—as job autonomy and social support—are “those physical, psychological, social, or organizational aspects of the job that are functional in achieving work goals, reduce JDs and the associated physiological and psychological costs, or stimulate personal growth, learning, and development” (Bakker and Demerouti, 2017, p. 274).

While high JDs deplete employees’ psychophysical resources and lead to strain, ill-health, and reduced job performance (JP), high JRs entail increased motivation and higher JP, which reflects the health-impairment and the motivational process of the JD-R, respectively (Schaufeli and Taris, 2014). With respect to the latter, JRs have motivational potential and stimulate WE either through the satisfaction of basic human needs or the achievement of one’s goals at work. Specifically, JRs may have either an intrinsic motivational role, since they promote employees’ growth and development through the satisfaction of basic human needs (Deci et al., 2017), or an extrinsic motivational role, because a resourceful work environment sustains workers’ willingness to devote one’s efforts and abilities to work tasks, thereby enhancing goal attainment (Schaufeli and Taris, 2014). In both cases, JRs are expected to promote WE.

Furthermore, building on the idea that work-related stress and well-being result from the interplay between personal and environmental factors (Hart and Cooper, 2001; Schaufeli and Taris, 2014; Karunamuni et al., 2021), the JD-R theory acknowledges the role of personal characteristics in the motivational/health-impairment processes, which is a second advantage of the theory. Specifically, the JD-R distinguishes between personal demands and personal resources (PRs), the latter referring to those “psychological characteristics or aspects of the self that are generally associated with resiliency and that refer to the ability to control and impact one’s environment successfully” (Schaufeli and Taris, 2014, p. 49). Like JRs, PRs seems to play a key role in the motivational process of the JD-R. First, PRs have a reciprocal relationship with JRs, so that workers with greater PRs may have access to more JRs, and vice versa (Bakker et al., 2023). Furthermore, by being functional in accomplishing work goals as well as stimulating personal growth and development (Schaufeli and Taris, 2014), PRs are expected to have a direct positive effect on WE (Bakker and Demerouti, 2017). Summarizing, according to the JD-R, both JRs and PRs jointly contribute to WE, with positive outcomes in terms of JP.

### 1.2 The current study

In the current study we examined SS and resilience as relevant JR and PR—respectively—that can promote WE over time. Supervisor support is related to the broader construct of social support, or the “interpersonal support from other individuals at work” (Jolly et al., 2021, p. 229). Although social support may come from a variety of sources (Jolly et al., 2021), including supervisors or colleagues, in this study we focused on general SS that involves “expressions of concern by the supervisor (i.e., emotional support) or tangible assistance (i.e., instrumental support) that is intended to enhance the well-being of the subordinate” (Kossek et al., 2011, p. 292). Specifically, one’s supervisor may offer understanding and encouragement in front of difficulties or when errors are made, while also complimenting employees for their achievements, but she/he may also provide assistance with work-related problems, heavy workload or difficult assignments (Bélanger et al., 2016; Gonzalez-Morales et al., 2018; Tews et al., 2020).

In this study the focus was on SS since support form one’s supervisor plays a pivotal role in the organizational life of workers (Ng and Sorensen, 2008; Ahmad et al., 2022), also because employees often interpret supervisors’ behaviors as representing their organizations (Stinglhamber and Vandenberghe, 2003). It has also been proposed that the supervisor-employee relationship is essential in energizing and motivating individuals to excel at work (Swanberg et al., 2011). Not surprisingly, a previous meta-analysis by Ng and Sorensen (2008) showed SS to be more strongly associated with job satisfaction, affective commitment—two constructs that are conceptually and empirically related to WE (Mazzetti et al., 2021)—as well as turnover intention compared to coworker support. Finally, knowing that one’s supervisor is available and responsive may be particularly useful in a risk environment (Tews et al., 2020), so the value of SS may be of particular importance during the COVID-19 crisis (Converso et al., 2021; Kim et al., 2022).

In line with the motivational process of the JD-R, SS seems to play a central role in fostering WE (Costa et al., 2015; De Carlo et al., 2020). There are several possible theoretical mechanisms underlying this association. For example, SS may lead to WE because it is functional for the effective completion of tasks and the achievement of goals at work: an employee may be helped by her/his supervisor to overcome temporary difficulties or to deal effectively with novel and challenging demands, which results in a successful completion of work tasks (Yürür and Sarikaya, 2012; Tews et al., 2020). Furthermore, by facilitating task accomplishment and skill development, instrumental aspects of SS may satisfy employees’ need for competence (i.e., the need to feel mastery over the work environment and to develop new skills; Van den Broeck et al., 2016). Similarly, the need for relatedness (i.e., the need to feel connected to other individuals) may be satisfied by emotional aspects of SS, which favors the development of a closer relationship with one’s supervisor and the integration in a social group (Jolly et al., 2021). Hence, by having both extrinsic and intrinsic motivational potential, SS is expected to promote WE. Accordingly, we hypothesized that SS at T1 will be positively associated with WE at T2.

*Hypothesis 1 (H1)*. Supervisor support at T1 will be positively associated with work engagement at T2.

Next, we focused on resilience. Defined by Stewart et al. (1997, p. 22) as “the capability of individuals to cope successfully in the face of significant change, adversity, or risk,” resilience refers to positive adjustment and thriving in the face of negative/adverse events such as crisis, stress or trauma (Aburn et al., 2016). Notably, as argued by Avey et al. (2009), resilience is probably the most important positive resource to navigate turbulent situations, including the COVID-19 crisis (Zhang et al., 2022). Not surprisingly, scholars’ and practitioners’ interest in workplace resilience has recently increased significantly in the field of work and organizational (WOP) psychology (Hartmann et al., 2020), with resilience being considered as one of the core constructs of positive organizational behavior (Luthans, 2002).

Building on the JD-R theory, WOP researchers often conceptualize resilience as a PR that can contribute to WE (Schaufeli and Taris, 2014). Specifically, there are several theoretical mechanisms through which resilience may play a role in the motivational process of the JD-R, thus leading to WE. For example, resilient employees might be better able to meet JDs and attain their goals at work, while also being capable to fulfill their basic psychological needs for competence by actively addressing demands or seeking new challenges at work (Lu et al., 2022). Similarly, resilient workers may have access to more JRs (e.g., opportunity for professional growth or career advancement; Bakker et al., 2023) and experience a greater ability to control and affect their environment successfully (Kašpárková et al., 2018), which may give rise to WE. Finally, by enabling individuals to effectively manage threats and problems at work, resilience may contribute to prevent burnout, a negative type of SWB (Lu et al., 2022). Summarizing, resilience may have motivational potential because it can help employees in achieving work-related goals, fulfilling basic human needs, and dealing with current JDs. From an empirical standpoint, past research has shown that employees’ resilience is related to well-being at work, in terms of reduced exhaustion/cynicism, job self−/supervisor-rating of performance, and, clearly, WE (Luthans et al., 2005; Hartmann et al., 2020; Mazzetti et al., 2021). Hence, based on both theoretical reasoning and empirical results, we hypothesized that resilience at T1 will be positively associated with WE at T2.

*Hypothesis 2 (H2)*. Resilience at T1 will be positively associated with work engagement at T2.

The last hypothesis concerns the focal association between WE and hair DHEA(S), considered in this study as a possible biomarker of employees’ well-being. As well as being a precursor to sex steroid, DHEA(S) is an anabolic steroid that plays a regenerative role in the body (Dutheil et al., 2021), improves physical well-being (Ahmed et al., 2023), and contributes to increasing motivation and overall well-being. Moreover, DHEA(S) affects neurosteroidogenis and endorphin synthesis or release (Pluchino et al., 2015). Biological actions of this hormone involve neuroprotection, neurite growth, neurogenesis, neuronal survival, apoptosis, catecholamine synthesis and secretion, as well as anti-inflammatory, anti-oxidant and antiglucocorticoid effects (Maninger et al., 2009; Pluchino et al., 2015; Whitham et al., 2020).

It has been suggested that DHEA(S), as an antagonist to the effects of cortisol, may have a protective role during acute stress (Lennartsson et al., 2013a), and empirical studies have shown serum concentrations of DHEA(S) to be lower among individuals reporting stress at work (Lennartsson et al., 2013b). Furthermore, DHEA(S) was found to be associated with subjective health and life satisfaction (Berr et al., 1996), while low DHEA(S) was observed among people in relatively poor health because of stressful events such as accident or surgery (Baulieu, 1996). Different studies have reported lowered serum concentrations of DHEA(S) to be associated with poor life satisfaction, psychosocial stress, functional limitations, and subjects more vulnerable, while higher plasma and serum concentrations have also been associated with better cognitive ability, greater amount/frequency/enjoyment of leisure activities and healthier psychological profiles (Pluchino et al., 2015).

Not surprisingly, DHEA(S) concentrations are generally considered a biomarker of physical and psychophysiological resilience and well-being (Russo et al., 2012; Rutkowski et al., 2014; Osório et al., 2017). However, previous research also showed DHEA(S)—in plasma, serum or saliva—to be related with vigor, positive affective states (e.g., enthusiasm), and job performance, but inversely associated with depressed mood and burnout (Barrett-Connor et al., 1999; Rasmusson et al., 2006; Morgan et al., 2009; Petros et al., 2013; Zarit et al., 2014; Lennartsson et al., 2015), all aspects related to WE (Schaufeli et al., 2002). Hence, building on theoretical considerations and empirical evidence, in this study we take a step further and we conceive DHEA(S) in hair as a potential biomarker of WE, the latest being a type of work-related subjective well-being that plays a central role in the motivational process of the JD-R theory (Bakker et al., 2023).

Particularly, we focused on hair measurement to detect the cumulative concentration of DHEA(S) between baseline and follow-up. The rationale for this choice is that, as noted by Bakker (2015, p. 842), the “repeated exposure to daily job resources will result in high levels of aggregated daily engagement, which predicts general work engagement” over time. Accordingly, we expect hair concentration of DHEA(S) to reflect the cumulative/sustained physiological activation that is associated with enduring, general WE—as a “persistent and pervasive affective-cognitive state” (Schaufeli et al., 2002, p. 74)—and that results from a prolonged experience of acute, short-term physiological activation associated with fluctuating WE states (e.g., at daily level), in response to JRs/PRs (Bakker, 2015). Stated differently, while DHEA(S) in serum/saliva is expected to reflect the short-term physiological activation associated with fluctuating WE states, hair DHEA(S) should reflect cumulative/sustained physiological activation associated with general, more persistent WE. We then hypothesized that WE at T2 will be associated with hair DHEA(S) at T2.

*Hypothesis 3 (H3)*: Work engagement at T2 will be positively associated with hair DHEA(S) at T2.

Hence, in line with this reasoning, our hypothesis is that SS/resilience at T1 will have a positive indirect effect on hair DHEA(S) at T2 through WE at T2, that is, WE will mediate the association between SS/resilience and DHEA(S).

*Hypothesis 4a (H4a)*. Work engagement at T2 will mediate the association between supervisor support at T1 and DHEA(S) at T2.

*Hypothesis 4b (H4b)*. Work engagement at T2 will mediate the association between resilience at T1 and DHEA(S) at T2.

## 2 Method

### 2.1 Sample

The study was conducted during the COVID-19 pandemic and involved workers from various organizations in Italy. The methodology of the study is described in detail elsewhere (Falco et al., 2023). Participants were recruited using snowball sampling. Briefly, participants were invited by members of the research team to take part in a longitudinal study about their work experience and hair biomarkers of stress and well-being. Upon acceptance, participants were given the opportunity to recommend other potential participants, who in turn were contacted by the researchers until an adequate sample size was reached. Data were collected at two different time points: Time 1 (T1, mid-March 2022) and Time 2 (T2, mid-June 2022), with a three-month time lag between waves. At both T1 and T2 participants were asked to complete an online self-report questionnaire to measure the focal psychological constructs. Participants were also informed that they would be contacted at T2 for the collection of a biological sample, that is, a strand of hair about 3 cm in length. Before completing the questionnaire at T1, all participants provided written informed consent, and they were also given an alphanumeric identification code. This code was necessary to match psychological data over time, as well as psychological and biological data. At T2, participants were provided with detailed instructions on how to collect biological samples. The project has been approved by the Ethical Committee for Psychological Research of the University of Padova.

All in all, 135 workers completed the online questionnaire at T1, and 122 (90.4%) also collected psychological and biological data at T2. No differences emerged in the main demographic and study variables at T1 between those who did or did not complete the study. The sample consisted of 90 women (73.8%) and 32 men (26.2%) with a mean age of 39.8 years (*SD* = 13.6). Fifty-one workers (41.8%) had less than 5 years of service in their current organization, whereas 47 (38.5%) had more than 10 years of service. Seventy-seven participants (63.1%) had a permanent contract, while 45 had a fixed-term one (36.9%). With respect to their occupation, 41 participants (33.6%) were white-collar, 27 (22.1%) were blue-collar, 26 (21.3%) were managers or self-employed, 14 (11.5%) were schools professionals, and 9 (7.4%) were healthcare professionals. Finally, most participants (57.4%) were married or cohabitating and had no children (59.8%).

### 2.2 Psychological measures

The self-report questionnaires comprised the following measures:

Work engagement was measured at T2 by using the Italian adaptation of the short version of the Utrecht Work Engagement Scale (i.e., UWES-9; Balducci et al., 2010). The scale comprised nine items designed to identify the three dimensions of WE, in terms of vigor (three items; e.g., “At my work, I feel bursting with energy”), dedication (three items; e.g., “I am enthusiastic about my job”), and absorption (three items; e.g., “I am immersed in my work”). In this study, the response scale ranged from 1 (*strongly disagree*) to 6 (*strongly agree*) and, consistent with the authors, an overall score of WE was used. Cronbach’s alpha was 0.89 for vigor, 0.92 for dedication, and 0.79 for absorption, while Cronbach’s alpha for the overall WE scale was 93.

Supervisor support was assessed at T1 by means of a scale (four items) taken from the Q_u_-Bo Test, an instrument standardized for the Italian context (De Carlo et al., 2008). A sample item is: “My supervisor helps me do my job to the best of my ability.” The response scale ranged from 1 (*strongly disagree*) to 6 (*strongly agree*), and higher scores reflected higher levels of SS. Cronbach’s alpha was 0.83 in this study.

Resilience was measured at T1 using a revised version of the Connor–Davidson Resilience Scale (CD-RISC; Connor and Davidson, 2003) in the Italian translation by Di Sipio et al. (2012). The authors adapted the scale to organizational and work contexts to better capture professional resilience, which is consistent with recommendations from the literature to explore the concept of resilience in specific population groups (i.e., working adults), given its contextual nature (Aburn et al., 2016). The scale comprised 10 items (e.g., “I can achieve work goals despite obstacles”). The response scale ranged from 1 (*strongly disagree*) to 6 (*strongly agree*), and higher scores reflected higher levels of resilience. Cronbach’s alpha was 0.87 in this study.

### 2.3 Biological measures

The hair was collected non-invasively and painlessly from the vertex posterior region of the head. Each sample was stored in a paper envelope at room temperature and protected from UV rays until processing. Twenty-five milligrams of hair were weighted, and each hair strand was washed twice using H2O for 3′ and then, in agreement with Davenport et al. (2006) twice with isopropanol for 3′. Steroids were extracted by incubating each specimen for 16 h in methanol at 37°C. Next, the liquid in the vial was evaporated to dryness at 37°C under an airstream suction hood. The dried residue was then re-suspended in 1.2 mL of ELISA buffer (50 mM phosphate buffer, pH 7.4, 0.4% BSA, 0.5 M NaCl). The concentrations of DHEA(S) were measured using an in-house Enzyme-linked Immunosorbent Assay (ELISA) as described by Falco et al. (2023).

### 2.4 Data analysis

First, the factor structure, construct validity, and reliability of the self-report instruments used in the study were examined by means of confirmatory factor analysis (CFA). The procedure is described in the Supplementary material.

Next, the hypothesized relationships were tested using structural equation modeling with observed variables, that is, path analysis. Specifically, a model was estimated in which DHEA(S) at T2 was the dependent variable, WE at T2 was the mediator, and SS/resilience at T1 were the independent variables. All the structural paths were estimated freely, to test direct and indirect effects simultaneously, which resulted in a just-identified path model. Confidence intervals for direct/indirect effects were derived via percentile bootstrap (10,000 resamples), which offers good performance across a variety of data conditions (Falk, 2018). A statistically significant direct or indirect (i.e., mediated) effect is supported if the corresponding confidence interval does not contain zero. The bootstrapping approach is especially useful to establish confidence intervals for the mediation effects, since the adoption of a significance test that assumes a normal distribution, when there is evidence that the distribution of mediation effect is not normal, may not be appropriate (Cheung and Lau, 2008). In addition, as previous research has shown an association between DHEA(s) concentrations and sex as well as age, the model was estimated controlling for the effect of these demographic variables (Petros et al., 2013; Lennartsson et al., 2013b; Qiao et al., 2017; Ahmed et al., 2023). Similarly, sex and age differences in work engagement are reported in the literature (Eurofound, 2022). Finally, a logarithmic transformation was applied to DHEA(S) to improve its distribution and symmetry (Becker et al., 2019).

For both CFA and path models the estimator was the maximum likelihood method (Rosseel, 2012). The chi-square test was used to assess model fit along with RMSEA, CFI, and SRMR. A model shows a good fit to data if χ^2^ is nonsignificant. Additionally, values close to or smaller than 0.08 for RMSEA and SRMR, as well as values close to or greater than 0.90 for CFI, indicate an acceptable fit (Brown, 2015). Statistical analyses were carried out using the lavaan package version 0.6–14 (Rosseel, 2012) for R software version 4.2.1.

## 3 Results

### 3.1 Confirmatory factor analysis

Good psychometric properties in terms of factor structure, convergent/discriminant validity and reliability were found for the self-report questionnaires used in this study. Results of the CFAs models are described in detail in the Supplementary material.

### 3.2 Descriptive statistics

Univariate skewness and kurtosis were within acceptable ranges for all variables (Finney and DiStefano, 2013). Correlations and descriptive statistics are presented in Table 1. Work engagement at T2 was positively associated with log DHEA(S) at T2 (*r* = 0.21, *p* = 0.02). Also, WE at T2 was positively associated with both SS at T1 (*r* = 0.29, *p* < 0.01) and resilience at T1 (*r* = 0.38, *p* < 0.001), with SS at T1 also being positively associated with log DHEA(S) at T2, although this association was marginally significant (*r* = 0.16, *p* = 0.09). With respect to control variables, there were no differences in log DHEA(S) or WE across sex, while a negative—albeit marginally significant—association between age and log DHEAS(s) emerged (*r* = −0.16, *p* = 0.09).

**Table 1:** Descriptive statistics and correlations for study variables (*N* = 122).

	*M*	*SD*	1	2	3	4	5	6
1. DHEA(S) pg./mg – T2	67.60	56.12	−					
2. Work engagement – T2	4.01	0.99	0.21^*^	(0.93)				
3. Sex – T1^a^	0.26	0.44	−0.01	−0.01	−			
4. Age – T1	39.76	13.59	−0.16^†^	0.09	−0.16^†^	−		
5. Supervisor support – T1	3.30	1.35	0.16^†^	0.29^**^	0.11	−0.21^*^	(0.83)	
6. Resilience – T1	4.03	0.84	0.04	0.38^***^	0.21^*^	−0.01	0.19^*^	(0.87)

DHEA(S) values were log-transformed prior to data analyses, including correlations shown above. Internal consistency of the scales (Cronbach’s alpha) is displayed in the diagonal of the correlation matrix within parentheses. DHEA(S) = dehydroepiandrosterone sulfate; T1 = Time 1; T2 = Time 2.

^a^0 = Female, 1 = male.

^†^*p* < 0.10, ^*^p < 0.05, ^**^p < 0.01, ^***^p < 0.001.

### 3.3 Hypothesis testing

The results of the path analysis model are shown in Table 2. In this model, SS at T1 was positively associated with WE at T2, controlling for the effect of sex, age and resilience, unstandardized β = 0.20, *p* < 0.01, 95% CI [0.08, 0.32]. Similarly, resilience at T1 was positively associated with WE at T2, controlling for the effect of sex, age and SS, unstandardized β = 0.41, *p* < 0.001, 95% CI [0.21, 0.60]. Therefore, H1 and H2 were supported. Furthermore, WE at T2 was positively associated with log DHEA(S) at T2 controlling for sex, age, and predictors at T1, unstandardized β = 0.07, *p* = 0.03, 95% CI [0.001, 0.13], and H3 was supported. The indirect effect of SS at T1 on log DHEA(S) at T2 through WE at T2 was positive and significant, unstandardized β = 0.01, 95% CI [0.001, 0.03]. Similarly, the indirect effect of resilience at T1 on log DHEA(S) at T2 through WE at T2 was positive and significant, unstandardized β = 0.03, 95% CI [0.001, 0.06]. Therefore, WE at T2 mediated the relationship between SS/resilience at T1 and log DHEA(S) at T2, and H4a and H4b were supported. Furthermore, neither SS nor resilience at T1 were associated with log DHEA(S) at T2, controlling for sex, age, and WE (Table 2 and Figure 1). Finally, an additional model in which the nonsignificant paths from SS/resilience at T1 to log DHEA(S) at T2 were fixed at zero was estimated to obtain a more parsimonious solution. As expected, the model showed a good fit to data: χ^2^(2) = 0.72, *p* = 0.70; RMSEA = 0, CFI = 1, SRMR = 0.014, and results were substantially unchanged (please see the Supplementary material). Overall, our findings suggest that SS and resilience at T1 may contribute to WE at T2, which, in its turn, is positively associated with hair DHEA(S) throughout the observation period, from baseline to follow-up.

**Table 2:** Associations between hair dehydroepiandrosterone sulfate, work engagement, supervisor support, and resilience (*N* = 122).

	Dependent variables (Time 2)							
	Dehydroepiandrosterone sulfate				Work engagement			
Direct effects	Estimate	*SE*	95% CI		Estimate	*SE*	95% CI	
			*LL*	*UL*			*LL*	*UL*
Work engagement (T2)	0.069^*^	0.031	0.001	0.133				
Sex^a^ (T1)	−0.023	0.064	−0.148	0.103	−0.192	0.186	−0.568	0.147
Age (T1)	−0.004	0.002	−0.008	0.001	0.010	0.006	−0.001	0.021
Supervisor support (T1)	0.016	0.022	−0.030	0.060	0.197^**^	0.061	0.075	0.322
Resilience (T1)	−0.017	0.036	−0.088	0.061	0.407^***^	0.098	0.206	0.603
Indirect effects								
Supervisor support→DHEA(S)	0.013	0.007	0.001	0.030				
Resilience→DHEA(S)	0.028	0.014	0.001	0.056				
Total *R*^2^	0.080				0.221			

Dehydroepiandrosterone sulfate values were log-transformed prior to data analyses. Confidence intervals were estimated using percentile bootstrap (10,000 samples). DHEA(S), dehydroepiandrosterone sulfate; SE, standard error; CI, confidence interval; LL, lower limit; UL, upper limit; T1, Time 1; T2, Time 2.

a 0 = Female, 1 = male.

^*^p < 0.05, ^**^p < 0.01, ^***^p < 0.001.

## 4 Discussion

Based on the motivational process of JD-R theory (Bakker et al., 2023), in the current study we examined the longitudinal associations between SS and resilience—as JRs and PRs, respectively—and WE during the COVID-19 pandemic. We also investigated whether DHEA(S) could be considered a biomarker of employees’ well-being based on its association—indirect or direct—with JRs/PRs and WE. The results revealed that SS and resilience at T1 were positively associated with WE at T2, which, in turn, was positively associated with hair DHEA(S) at T2, the latter reflecting cumulative biomarker concentrations throughout the observation period, from baseline to follow-up (Marceau et al., 2021). Additionally, our study showed that SS and resilience at T1 had a positive, indirect effect on DHEA(S) at T2 through WE at T2. These findings suggest that both SS and resilience may have a significant impact in promoting employee engagement at work and the associated physiological response (Osório et al., 2017; Mazzetti et al., 2021). Furthermore, our study provides initial evidence for the role of hair DHEA(S) as a biomarker of WE, a type of work-related subjective well-being that plays a central role in the motivational process of the JD-R theory, leading to favorable personal and organizational outcomes (Bakker et al., 2023).

All in all, we believe that our study makes a valuable contribution to the field. First, our results on the association between WE and DHEA(S) are relevant and quite original, compared to previous research. For example, Langelaan et al. (2006) conducted a study on a sample of managers from a telecommunications company and found that the burnout, engaged and reference groups did not differ in salivary DHEA(S) levels. Similarly, a recent cross-sectional study showed no association between WE and hair DHEA among women employees in a medical services company (Wacker et al., 2021). Interestingly, Lennartsson et al. (2015) investigated serum concentration of DHEA(S) among patients with burnout, which has opposite characteristics to WE (Oerlemans and Bakker, 2011). This study has shown serum concentrations of DHEA(S) to be lower among younger burnout patients (25–35 years) compared to healthy controls, with this difference being more pronounced among female than among male participants. However, no differences emerged between patients and controls in the other age groups (36–45 and 46–54 years).

Given the complex picture that emerges from past research, there are two main features of our study that need to be acknowledged. First, most previous research relied on assessment of biomarkers in saliva/serum that reflect acute, short-term physiological activation (Dutheil et al., 2021). Contrarily, our study focused on hair DHEA(S), which reflects the cumulative/sustained physiological activation associated with general, more persistent WE, which is consistent with recent conceptualizations of the construct (Bakker, 2015; Bakker et al., 2023). Second, while previous research has been conducted in specific populations (e.g., managers, patients with clinical burnout), our study aimed to investigate the hypothesized associations in the general working population.

As an additional contribution, our study showed that SS and resilience—as JRs and PRs—play a central role in promoting well-being and, specifically, WE. In fact, both SS and resilience were positively associated with WE, but also indirectly related to DHEA(S), a pattern of results which is consistent with the motivational process of JD-R theory. Our findings support past research indicating that SS and resilience are important factors in fostering WE (Mazzetti et al., 2021; Kossyva et al., 2022), particularly during the COVID-19 crisis (Hajjami and Crocco, 2023). For example, a recent literature review conducted amidst the COVID-19 pandemic (Hajjami and Crocco, 2023) revealed that supervisors can support employees by promoting transparent and timely communication, as well as building cooperation and trust (e.g., through daily face-to-face/virtual huddles; Sinclair et al., 2021), which can ultimately lead to greater WE. Furthermore, by providing explicit guidance and adopting compassionate behavior, supervisors can maintain the connection between workers and their organization as well as promote support among team members during COVID-19 pandemic, with a positive impact on WE (Hajjami and Crocco, 2023).

Similarly, our study showed that resilience is a main driver of WE, which is consistent with previous research (Mazzetti 2022; 10.1108/MRR-01-2021-0043). It is not surprising that resilience is particularly relevant during the COVID-19 pandemic (Killgore et al., 2020). Highly resilient employees have many positive characteristics that contribute to WE, including optimism and energy, which is particularly important in time of crisis including the COVID-19 pandemic (Blaique et al., 2022). Additionally, by being able to successfully control their environment, resilient individuals are intrinsically motivated to pursue their goals and better able to cope with adversity and changes (Malik and Garg, 2020). Furthermore, a previous study conducted during the COVID-19 pandemic suggested that employees with high levels of confidence in their ability to perform job tasks (i.e., high self-efficacy) were more able to develop resilience and to recover more quickly from work-related stress, potentially leading to greater WE (Ojo et al., 2021).

Taken together, this study shed light on the organizational and personal driver of WE during the COVID-19 pandemic, and provides new insight into the physiological processes involved in motivational processes in JD-R theory (Bakker and Demerouti, 2017). Given the paucity of research and the heterogeneity of previous results, this study outlines interesting avenues for future research on possible biomarkers of WE as a type of work-related subjective well-being that plays a central role in the motivational process of the JD-R theory, leading to favorable personal and organizational outcomes (Bakker et al., 2023).

### 4.1 Limitations

There are some limitations to the current study that need to be acknowledged. Although the current study met the recommended minimum sample size of 100 participants (Bagozzi, 2010), the sample size is small and the sex distribution (26.2% male) and other demographics are imbalanced. Although sex imbalance is not uncommon in studies of biomarkers in hair (e.g., those who are bald or have hair less than 3 cm long cannot participate; Ben Assayag et al., 2017), the aforementioned limitation may limit the generalizability of the findings to a broader population. To obtain more accurate and widely applicable results, larger sample sizes may be required in future research endeavors to enhance statistical power as well as replicate and extend present findings. Second, the study’s time frame was limited to a relatively short period, with a three-month time lag between waves (baseline and follow-up), which may not sufficiently capture the long-term effects of support from supervisor, resilience, and work engagement on DHEA(S) levels among workers. However, the decision to use a 3-month time interval is based on both psychological and biological considerations. On the one hand, it has been proposed that both favorable and stressful situations at work (e.g., JRs and JDs, respectively) may lose their impact on individuals’ health and well-being in three months or less (Diener, 2000; Huyghebaert et al., 2018). On the other hand, measurement of steroid hormones in hair has been proposed as a useful method for research to describe long-term retrospective endogenous steroid hormone concentrations, with a lag-time of 1 month per centimeter of hair growth. The dosage of hormones in the hair reflects their average concentration over months because endo- and exogenous compounds are continuously incorporated from blood to hair follicles during hair growth. In addition, hormones captured inside the hair are stable (Eisenbeiss et al., 2020; Peng et al., 2022). Furthermore, common practice in hair analysis is to cut and analyze the most proximal three centimeters of hair collected noninvasively from the vertex posterior of the head of the scalp (Preinbergs et al., 2023). Third, the reliance on self-reported measures of the study variables may be limited by the response and social desirability biases. Thus, caution is needed when interpreting the current findings. A fourth limitation of this study is its correlational nature, which precludes the establishment of causal relationships between variables, such as supervisor support, resilience, and WE. Without manipulation or control of extraneous variables, it is difficult to ascertain whether changes in one variable (e.g., supervisor support) cause changes in another variable (e.g., work engagement). Therefore, the findings should be interpreted as associations rather than causality. Fifth, we focused exclusively on SS and resilience as antecedents, but other JRs—both physical and psychosocial, such as for example restorativeness of the work environment and job autonomy—and PRs (e.g., self-efficacy, optimism, and mindfulness; Luthans, 2002; Schaufeli and Taris, 2014; Ramaci et al., 2020; Bellini et al., 2022) are known to play a role in the motivational process of the JD-R (Mazzetti et al., 2021). Furthermore, according to the boost hypothesis of the JD-R theory, JDs amplify the impact of JRs on WE (Bakker et al., 2023). Hence, future research could investigate whether JDs, such as work complexity or workload, may strengthen the longitudinal associations between JRs/PRs and WE/DHEA(S). Finally, the role of personal demands, for example in terms of perfectionism or workaholism, could be further investigated (Girardi et al., 2015, 2018; Zeijen et al., 2021). All in all, due to these potential limitations, additional research is required to replicate and expand upon the current preliminary findings.

### 4.2 Contribution

Despite the above-mentioned limitations, this study provides valuable insights into the longitudinal associations between SS, resilience, WE, and hair DHEA(S), and highlights the importance of addressing these factors in workplace stress management and health promotion programs. The findings presented in this paper provide significant theoretical and practical contributions. From a theoretical standpoint, our study contributes to the theoretical understanding of the underlying mechanisms through which SS, resilience, and WE impact the psycho-physiological mechanisms involved in stress regulation and employees’ psychological health. By showing that WE at T2 fully mediated the association between SS/resilience at T1 and hair DHEA(S) at T2, this study supports the notion that WE is a vital component in the promotion of employees’ well-being and related positive psychological outcomes, capable of conveying the effect of psychological (e.g., resilience) and social (e.g., SS) resources. The findings also suggest that SS and resilience have an indirect effect on hair DHEA(S) levels through their influence on WE, highlighting the importance of considering multiple pathways in understanding the links between psychosocial factors and health outcomes.

The current findings also have important practical implications for interventions aimed at improving engagement and promoting well-being at work. Interventions that target improving SS and resilience can have a positive impact on WE and, in turn, promote better regulation of the stress response systems. Also, interventions focused on promoting well-being at work, such as resilience- and support-based stress reduction interventions, can also have a valuable significant impact on the regulation of the stress response systems at work. Understanding the mechanisms through which SS, resilience, and WE influence biological processes can facilitate the tailoring of effective interventions aiming at reducing stress-related outcomes at work.

Collectively, the findings of this study enhance our understanding of the complex relationship between psychosocial factors and biological processes involved in wellbeing and have significant implications for the development of interventions aimed at promoting psychological health and reducing stress-related outcomes at work, particularly for workers at risk of poor WE. For example, to sustain employees’ WE, training interventions could be targeted at supervisors, with the aim of strengthening their ability to recognize the specific needs of employees and to provide both instrumental and emotional support (i.e., primary prevention; Bakker et al., 2023). Additionally, in terms of secondary prevention, employees may be encouraged to proactively increase their social resources by changing the quality and/or quantity of social interactions with one’s supervisor at work (i.e., job crafting; Tims et al., 2021). Finally, training interventions could be aimed at promoting employee resilience, for example by strengthening their efficacy beliefs, learning and coping skills (Rodríguez-Sánchez, 2021).

## 5 Conclusion

In conclusion, this study investigated the associations between SS, resilience, WE and hair DHEA(S) over 3 months time period. The findings supported the hypotheses that SS and resilience at T1 were positively associated with WE at T2, and that WE at T2 was positively associated with hair DHEA(S) at T2. The study also found that SS and resilience at T1 had a positive indirect effect on hair DHEA(S) at T2 through WE at T2, which fully mediated the association between JRs/PRs and hair DHEA(S). These findings provide better insights into the complex interplay between psychosocial factors, WE, as well as physiological outcomes, and underscore the potential benefits of promoting positive workplace environments. Practically, the study highlights the importance of support from supervisors and resilience interventions in the workplace to enhance long-term effects on employee well-being and potentially improve physical health outcomes. Accordingly, multilevel interventions aimed at fostering WE should be targeted at both the leader/team and individual level, focusing on supervisors and employees, respectively (Nielsen et al., 2018). Finally, the current study gives initial evidence for hair DHEA(S) as a biomarker of WE, a type of work-related subjective well-being that plays a central part in the motivational process of the JD-R theory. Although further research is certainly needed, the measurement of hair DHEA(S) may prove a useful tool to early detect employees’ well-being, thus helping organizations and practitioners to promote health and performance at work. Certainly, organizations must be aware of the costs associated with analyzing biological samples. However, as the other side of the coin, we note that hair DHEA(S) assessment—as a non-invasive method that can be carried out by the workers themselves—can easily be extended to remote workers, with the aim of promoting inclusive and sustainable working conditions for all.

## Data availability statement

The raw data supporting the conclusions of this article will be made available by the authors, without undue reservation.

## Ethics statement

The studies involving humans were approved by Ethical Committee for Psychological Research of the University of Padova. The studies were conducted in accordance with the local legislation and institutional requirements. The participants provided their written informed consent to participate in this study.

## Author contributions

DG: Writing – review & editing, Writing – original draft, Investigation, Formal analysis, Conceptualization. LD: Writing – review & editing, Resources, Funding acquisition, Conceptualization. EA: Writing – review & editing, Writing – original draft, Visualization. MY: Writing – review & editing, Writing – original draft. IP: Writing – original draft, Resources. AP: Writing – original draft, Resources. AF: Writing – review & editing, Writing – original draft, Resources, Investigation, Conceptualization.
