# Supplementary material for: Hair dehydroepiandrosterone sulfate as biomarker of employees’ well-being? A longitudinal investigation of support, resilience, and work engagement during COVID-19 pandemic
Source: Front Psychol. 2024 Mar 20;15:1337839. doi: 10.3389/fpsyg.2024.1337839 (PMC10987734; doi:10.3389/fpsyg.2024.1337839)
Supplement: Supplementary file 1 [file Table_1.pdf]

Supplementary Material

1 Data Analysis

A confirmatory factor analysis (CFA) was carried out to evaluate the psychometric properties of the self-report instruments administered in the study, in terms of factor structure, construct validity, and reliability. In this model, each dimension of work engagement (WE), in terms of vigor, dedication, and absorption, as well as supervisor support (SS) were measured by the respective items, whereas resilience was measured by three parcels of scale items (Little et al., 2002). Construct validity—in terms of convergent and discriminant validity—and reliability were assessed using the average variance extracted (AVE) (Fornell and Larcker, 1981) and composite reliability (CR) (Bagozzi and Yi, 2012), respectively. An AVE greater than 0.50 indicates adequate convergent validity, and discriminant validity is established if the AVE of any two constructs is greater than their squared correlation, reflecting their shared variance (Fornell and Larcker, 1981). Values of CR greater than 0.70 suggest satisfactory reliability (Bagozzi and Yi, 2012).

Next, the hypothesized relationships were tested using structural equation modeling with observed variables, that is, path analysis. Specifically, a model was estimated in which dehydroepiandrosterone sulfate, or DHEA(S), at Time 2 (T2) was the dependent variable, work engagement at T2 was the mediator, and SS/resilience at Time 1 (T1) were the independent variables. In this model, the structural paths were freely estimated, to test direct and indirect effects simultaneously, except for the paths from SS/resilience at T1 to DHEA(S) at T2, which were nonsignificant in the model described in the manuscript. These paths were now fixed to zero. Confidence intervals for both direct and indirect effects were derived via percentile bootstrap (10,000 resamples), which offers good performance across a variety of data conditions (Falk, 2018). As in the manuscript, the model was estimated controlling for the effect of sex and age. Finally, a logarithmic transformation was applied to DHEA(S) to improve its distribution and symmetry (Becker et al., 2019).

For both CFA and path models the estimator was the maximum likelihood method (Rosseel, 2012). To assess model fit, the chi-square test was used along with RMSEA, CFI, and SRMR. A model shows a good fit to data if χ^2 is nonsignificant. Additionally, values close to or smaller than 0.08 for RMSEA and SRMR, as well as values close to or greater than 0.90 for CFI, indicate an acceptable fit (Brown, 2015). Statistical analyses were carried out using the lavaan package version 0.6–14 (Rosseel, 2012) for R software version 4.2.1.

2 Results

2.1 Confirmatory Factor Analysis

The hypothesized CFA model showed an acceptable fit to data: $\chi^2(94) = 177.51$, $p < 0.001$; RMSEA = 0.085, CFI = 0.937, SRMR = 0.079. The AVE ranged from 0.59 (absorption at T2) to 0.79 (dedication at T2), and AVE for each pair of latent factors was greater than their squared correlation. Additionally, CR ranged from 0.82 (absorption at T2) to 0.92 (dedication at T2). Overall, the self-report questionnaires used in this study showed good psychometric properties in terms of factor structure, convergent/discriminant validity, and reliability.

2.2 Hypothesis testing

To obtain a more parsimonious solution, an additional model was estimated, in which the nonsignificant paths from SS/resilience at T1 to log DHEA(S) at T2 were fixed to zero. As expected, the model showed a good fit to data: $\chi^2(2) = 0.72$, $p = 0.70$; RMSEA = 0, CFI = 1, SRMR = 0.014. Results were substantially unchanged, with SS at T1 being positively associated with WE at T2, controlling for the effect of sex, age and resilience, unstandardized $\beta = 0.20$, $p < 0.01$, 95% CI [0.08, 0.32]. Similarly, resilience at T1 was positively associated with WE at T2, controlling for the effect of sex, age and SS, unstandardized $\beta = 0.41$, $p < 0.001$, 95% CI [0.21, 0.60], and WE at T2 was positively associated with log DHEA(S) at T2 controlling for sex and age, unstandardized $\beta = 0.07$, $p = 0.01$, 95% CI [0.02, 0.13]. Finally, the indirect effect of SS at T1 on log DHEA(S) at T2 through WE at T2 was positive and significant, unstandardized $\beta = 0.01$, 95% CI [0.002, 0.03], as well as the indirect effect of resilience at T1 on log DHEA(S) at T2 through WE at T2, unstandardized $\beta = 0.03$, 95% CI [0.01, 0.05]. Overall, these results suggested that SS and resilience at T1 may contribute to WE at T2, which, in its turn, is positively associated with hair DHEA(S) during the observation period, between baseline and follow-up.

3 References

Bagozzi, R. P., and Yi, Y. (2012). Specification, evaluation, and interpretation of structural equation models. *J. Acad. Mark. Sci.* 40, 8–34. doi: 10.1007/s11747-011-0278-x.

Becker, T. E., Robertson, M. M., and Vandenberg, R. J. (2019). Nonlinear transformations in organizational research: Possible problems and potential solutions. *Organ. Res. Methods* 22, 831–866. doi: 10.1177/1094428118775205.

Brown, T. A. (2015). *Confirmatory factor analysis for applied research*. 2nd ed. New York, NY: Guilford Press.

Falk, C. F. (2018). Are robust standard errors the best approach for interval estimation with nonnormal data in structural equation modeling? *Struct. Equ. Model.* 25, 244–266. doi: 10.1080/10705511.2017.1367254.

Fornell, C., and Larcker, D. F. (1981). Evaluating structural equation models with unobservable variables and measurement error. *J. Mark. Res*. 18, 39–50. doi: 10.2307/3151312.

Little, T. D., Cunningham, W. A., Shahar, G., and Widaman, K. F. (2002). To parcel or not to parcel: Exploring the question, weighing the merits. *Struct. Equ. Model*. 9, 151–173. doi: 10.1207/S15328007SEM0902_1.

Rosseel, Y. (2012). lavaan: An R package for structural equation modeling. *J. Stat. Softw*. 48, 1–36. doi: 10.18637/jss.v048.i02.
